# Amende Honorable

**Published:** 1869-08-15

**Authors:** 


### *Amende Honorable.*

We sincerely regret that, by a most unaccountable inadvertency, there was allowed to appear in the number of of THE JOURNAL for July 1st, a letter over the signature of George R. Welding, M.D., reflecting upon Mr. S. S. White and Dr. Geo. J. Ziegler, of Philadelphia, respectively the publisher and one of the editors of our highly esteemed and valuable contemporary, the *Dental Cosmos.*

We believe that the communication referred to is both false in statement and calumnious in design, and most heartily beg pardon of our readers for its infliction upon them.

The *Dental Cosmos* is highly creditable to its publisher, Mr. White, and to the dental profession; and Dr. Ziegler discharges the duties of his editorial position with rare fidelity, industry and excellence.

